# Supplementary material for: Plant-expressed virus-like particles reveal the intricate maturation process of a eukaryotic virus
Source: Commun Biol. 2021 May 24;4:619. doi: 10.1038/s42003-021-02134-w (PMC8144610; doi:10.1038/s42003-021-02134-w)
Supplement: Supplementary file 2 — Supplementary Information [file 42003_2021_2134_MOESM2_ESM.pdf]

Supplementary Information

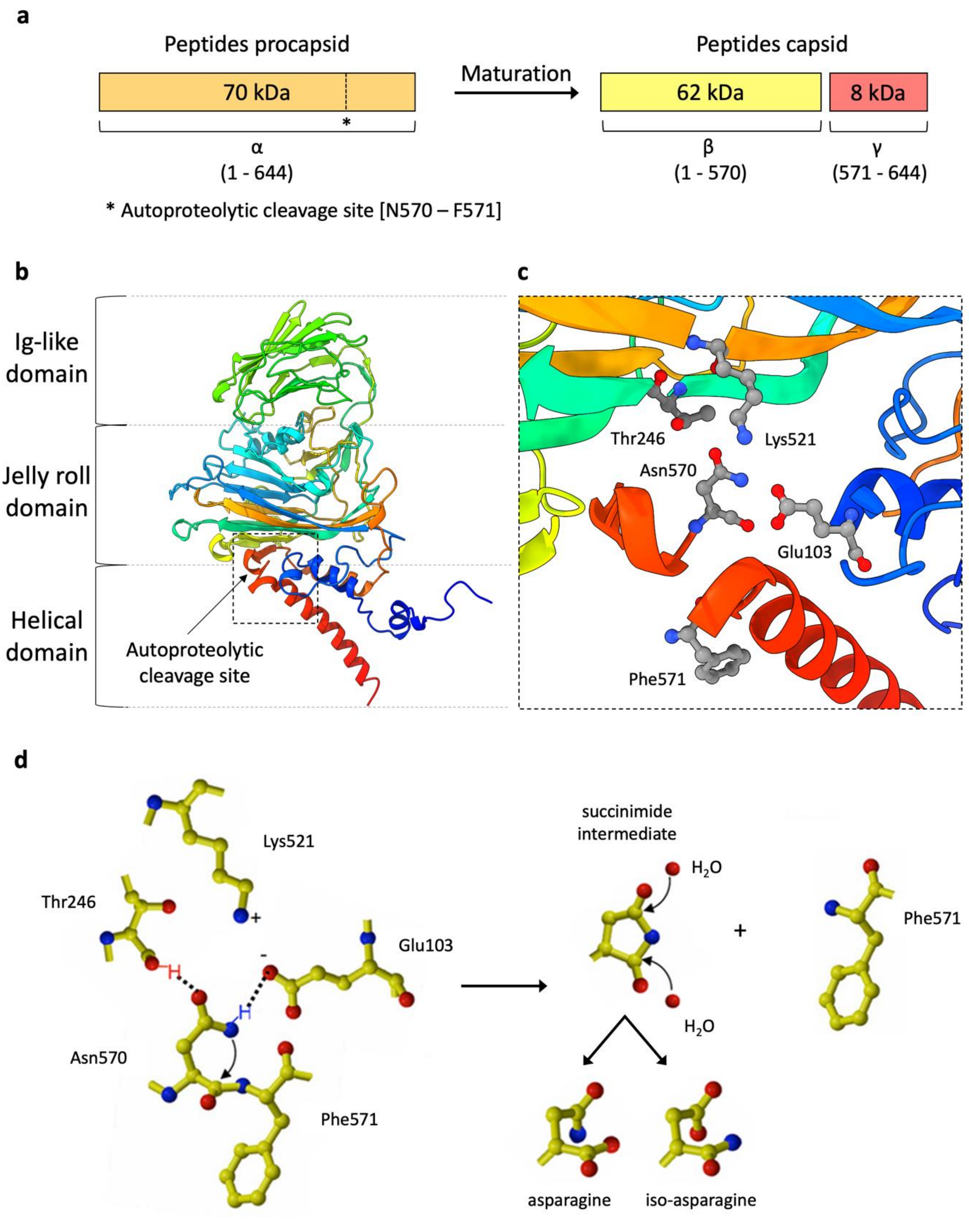

Figure S1: NωV maturation. (a) Cleavage of the coat protein during maturation. Schematic representation of the α protein and the cleavage site between residues N570 and F571. The subunit cleavage starts at pH 5.5 and generates the β peptide, that maintains the structure of the virus, and the γ peptide, which stays in contact with the capsid and is able to lyse cell membranes. **(b)** Ribbon representation of NωV capsid coat protein and autoproteolytic cleavage site showing a side view of the

A subunit. The rainbow colouring of the protein starts with blue at the N-terminus and ends with red at the C-terminus and the three domains within the subunit are indicated. **(c)** Cleavage site for processing of the α protein. To form the cleavage site, Glu103 and Thr246 or Lys521 form hydrogen bonds with the side chain of Asn570 to move it to the optimal location for autoproteolytic cleavage[1]. In the active site, the catalytic dyad consists of Asn570 and Glu103. This type of proteolytic enzyme is classified as an asparagine peptide lyase[2]. Structure obtained from PDB entry 1OHF[3,4]. **(d)** Proposed cleavage mechanism[1] based on the known degradation pathways in eye lens proteins[5].

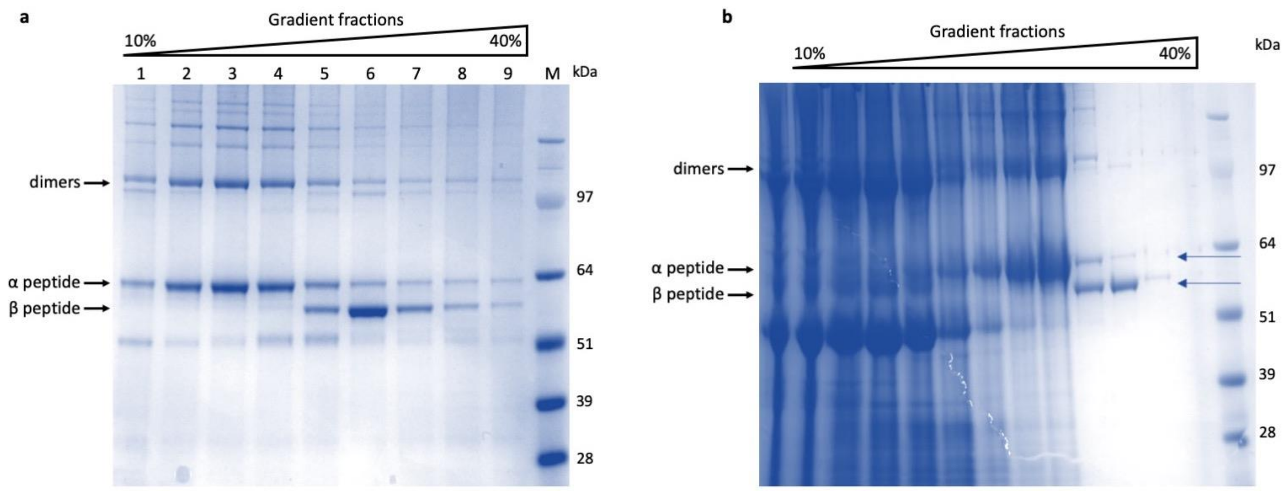

Figure S2: Sucrose (a) and (b) Optiprep gradients of VLPs extracted from plants.
Samples from the fractionated gradients were analysed by SDS-PAGE and fractions containing procapsids (a) and capsids (b) pooled. In (b), although the gradient was 10-50% Optiprep, only the fractions from 10-40% were analysed by SDS-PAGE.

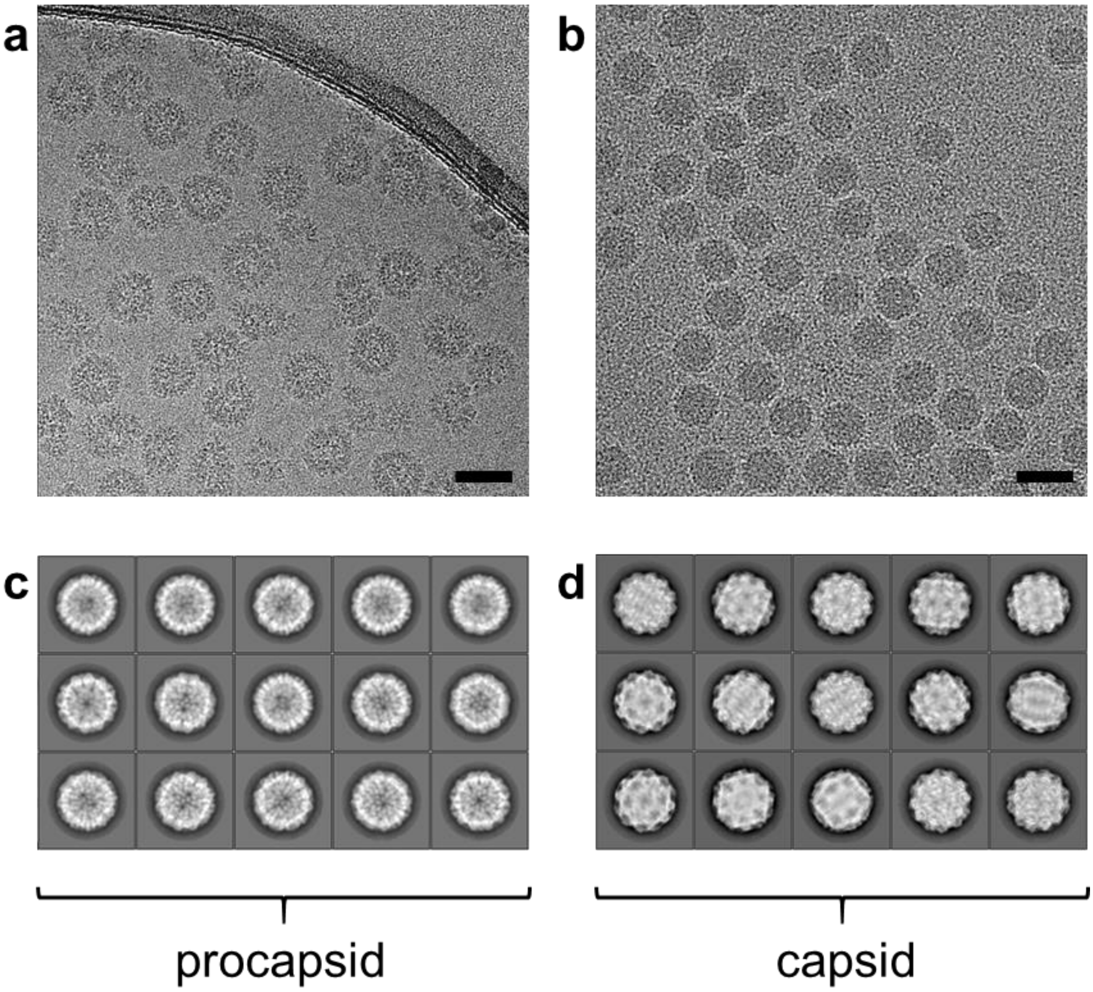

Figure S3: Cryo-EM data collection and processing. Motion-corrected movies (MOTIONCOR2) showing **(a)** procapsid and **(b)** capsid particles imaged in vitreous ice. Note that many of the procapsid particles are broken or deformed thereby significantly reducing the number of useable particles for reconstruction. The scale bar = 50 nm. 2D class averages (Relion) from the **(c)** procapsid and **(d)** capsid datasets (not to same scale).

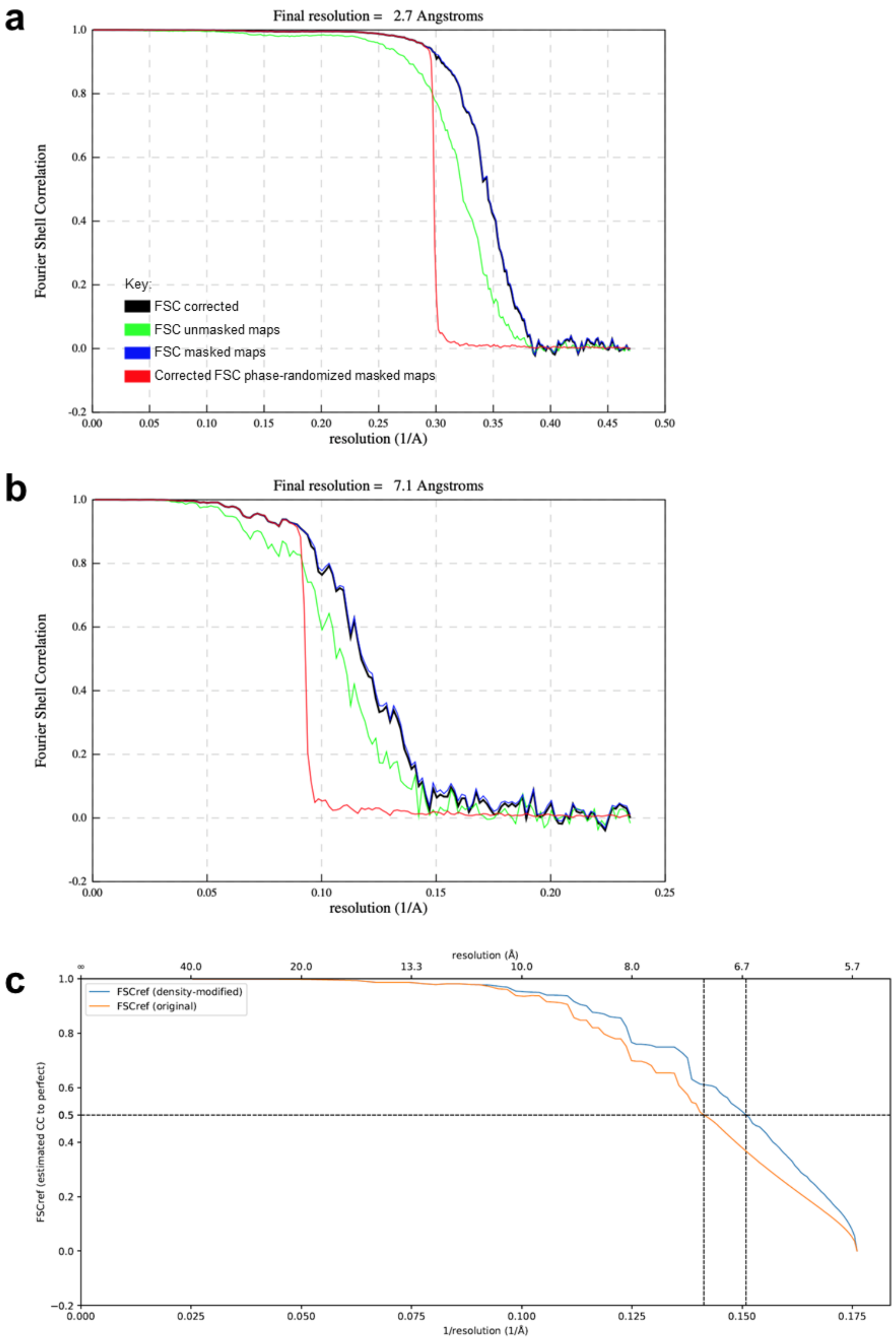

Figure S4: Cryo-EM reconstruction resolution estimation. Fourier Shell Correlation curves generated in the postprocessing step of Relion for **(a)** capsid and **(b)** procapsid reconstructions. The final resolutions were assessed using FSC = 0.143. The resolution of the latter was subsequently increased to 6.6 Å through density improvement as indicated by the FSC curves shown in **(c)** (see Methods).

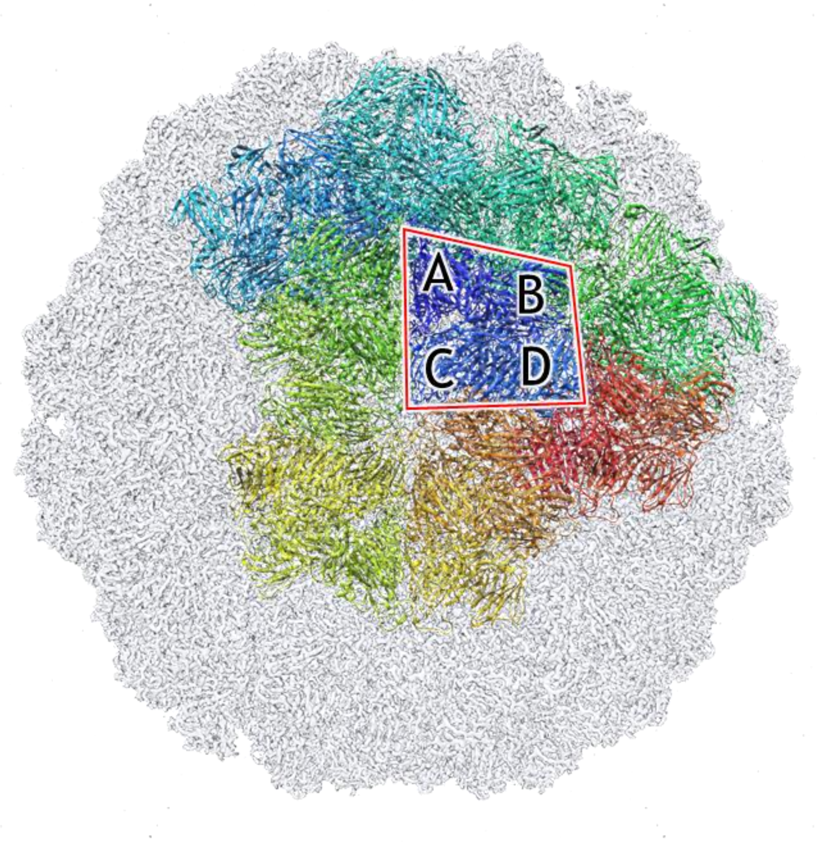

Figure S5: Cryo-EM model building and refinement. For convenience, rather than working with the full capsid or procapsid for visualization, model building and refinement, a subsection of the particle was defined as the asymmetric unit (ASU; outlined in red with subunits labelled) plus its eight nearest symmetry copies, denoted ASU8. Here ASU8 for the capsid is shown in ribbon representation (with each copy of the ASU in a different colour) in the context of the full sharpened map depicted in semi-transparent grey. Maps were cropped to ASU8 plus a 15 Å border, which significantly reduced the file size for the capsid map from 824 MB to only 48 MB and gave a corresponding reduction in the time taken for refinement jobs.

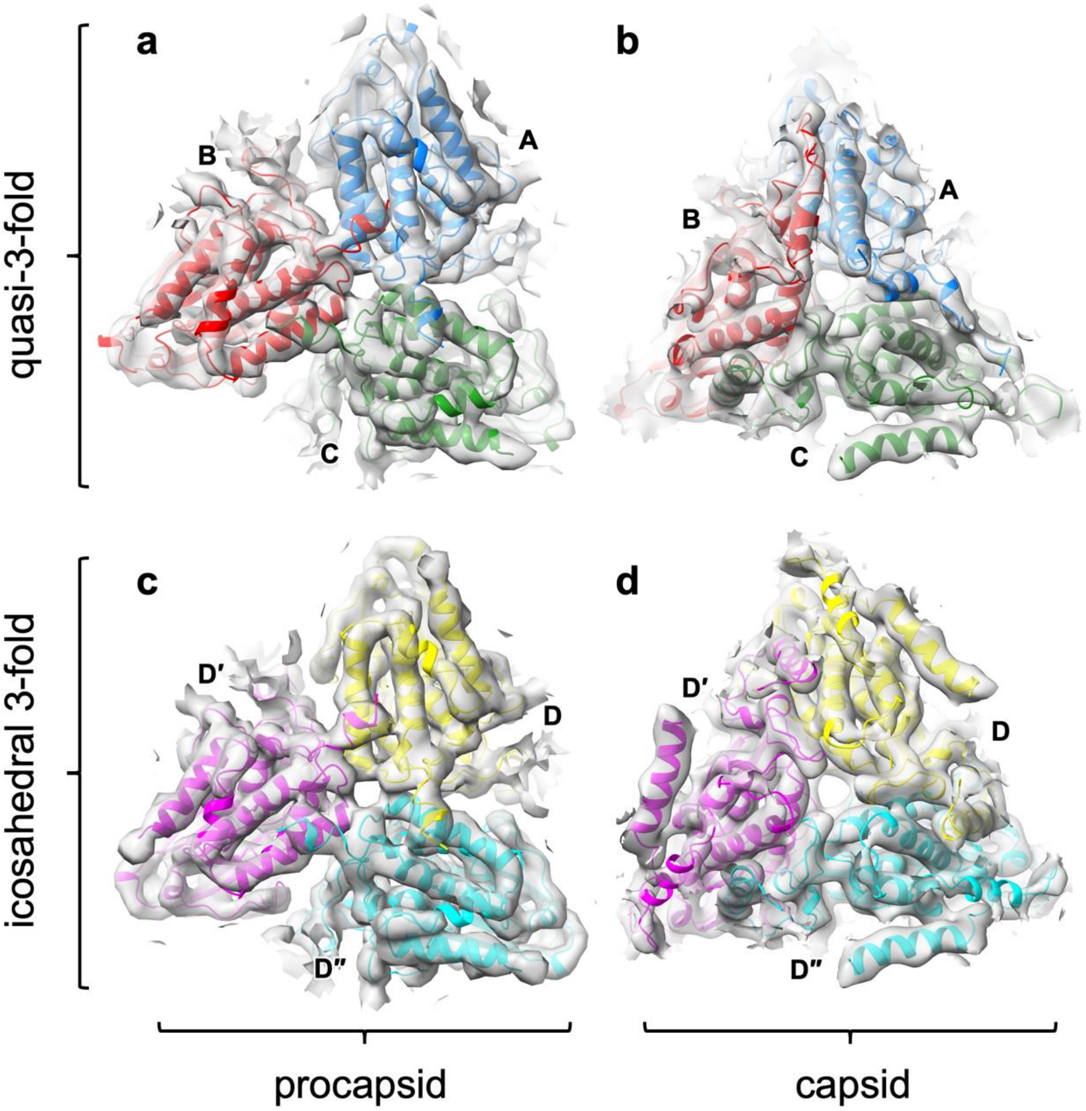

Figure S6: Comparisons of cryo-EM densities around the quasi-three-fold and icosahedral three-fold axes of capsid and procapsid reconstructions. Densities and fitted models around the quasi-three-fold axes for **(a)** the capsid and **(b)** the procapsid, and the icosahedral three-fold axes for **(c)** the procapsid and **(d)** the capsid as viewed from the inside. The subunit colouring scheme mirrors that used for the densities shown in Fig. 5 and again the capsid density has been down-sampled to 6.6 Å resolution. The three-folds have been aligned to facilitate comparison. It is clear that the subunit structure and arrangement are very similar for both axes in the procapsid, whilst they are distinctly different for the capsid. Nevertheless, there is some similarity between A and B and between C and D in the capsid (actually easier to compare C with D″; see the rmsd values reported in Table S2).

Table S1. Structural comparisons

Rmsd (Å)		capsid				procapsid
	subunit	A	B	C	D	A
capsid	A	0	0.559 (527)	0.756 (500)	0.978 (501)	1.560 (415)
	B		0	0.753 (500)	0.924 (503)	1.616 (465)
	C			0	0.668 (591)	1.831 (473)
	D				0	2.149 (474)
procapsid	A					0

Rmsd values for pairwise superpositions of subunits from the NwV capsid and procapsid structures. For the latter, non-crystallographic symmetry constraints were imposed during refinement; thus all subunits are equivalent and only A is shown. The values in brackets indicate the numbers of residues that were aligned.

Supplementary references

1. Taylor, D. J. & Johnson, J. E. Folding and particle assembly are disrupted by single-point mutations near the autocatalytic cleavage site of Nudaurelia capensis ω virus capsid protein. Protein Science 14, 401–408 (2005).

2. Rawlings, N. D., Barrett, A. J. & Bateman, A. Asparagine peptide lyases: A seventh catalytic type of proteolytic enzymes. Journal of Biological Chemistry 286, 38321–38328 (2011).

3. Helgstrand, C., Munshi, S., Johnson, J. E. & Liljas, L. The refined structure of Nudaurelia capensis ω Virus reveals control elements for a T = 4 capsid maturation. Virology 318, 192–203 (2004).

4. Munshi, S. et al. The 2.8 Å structure of a T=4 Animal virus and its implications for membrane translocation of RNA. Journal of Molecular Biology 261, 1–10 (1996).

5. Stephenson, R. C. & Clarke, S. Succinimide Formation from Aspartyl and Asparaginyl peptides as Model for the Spontaneous Degradation of Proteins. Journal of Biological Chemistry 264, 6164–6170 (1989).
