# Supplementary material for: Plant-expressed virus-like particles reveal the intricate maturation process of a eukaryotic virus
Source: Commun Biol. 2021 May 24;4:619. doi: 10.1038/s42003-021-02134-w (PMC8144610; doi:10.1038/s42003-021-02134-w)
Supplement: Supplementary file 3 — Description of Additional Supplementary Files [file 42003_2021_2134_MOESM3_ESM.pdf]

Description of Additional Supplementary Files

File name: Supplementary Data 1

Description: Raw data of protein maturation shown in Figure 2.

File name: Supplementary Data 2

Description: Raw data on pH dependence of liposome lysis.
