# Supplementary material for: Plant-expressed virus-like particles reveal the intricate maturation process of a eukaryotic virus
Source: Commun Biol. 2021 May 24;4:619. doi: 10.1038/s42003-021-02134-w (PMC8144610; doi:10.1038/s42003-021-02134-w)
Supplement: Supplementary file 6 — Reporting Summary [file 42003_2021_2134_MOESM6_ESM.pdf]

nature research

Corresponding author(s): G.P Lomonossoff

Last updated by author(s): Apr 5, 2021

Reporting Summary

Nature Research wishes to improve the reproducibility of the work that we publish. This form provides structure for consistency and transparency in reporting. For further information on Nature Research policies, see our Editorial Policies and the Editorial Policy Checklist.

Statistics

For all statistical analyses, confirm that the following items are present in the figure legend, table legend, main text, or Methods section.

n/a	Confirmed	
[ ]	[x]	The exact sample size (*n*) for each experimental group/condition, given as a discrete number and unit of measurement
[ ]	[x]	A statement on whether measurements were taken from distinct samples or whether the same sample was measured repeatedly
[ ]	[x]	The statistical test(s) used AND whether they are one- or two-sided<br>*Only common tests should be described solely by name; describe more complex techniques in the Methods section.*
[x]	[ ]	A description of all covariates tested
[x]	[ ]	A description of any assumptions or corrections, such as tests of normality and adjustment for multiple comparisons
[x]	[ ]	A full description of the statistical parameters including central tendency (e.g. means) or other basic estimates (e.g. regression coefficient) AND variation (e.g. standard deviation) or associated estimates of uncertainty (e.g. confidence intervals)
[x]	[ ]	For null hypothesis testing, the test statistic (e.g. *F*, *t*, *r*) with confidence intervals, effect sizes, degrees of freedom and *P* value noted<br>*Give P values as exact values whenever suitable.*
[x]	[ ]	For Bayesian analysis, information on the choice of priors and Markov chain Monte Carlo settings
[x]	[ ]	For hierarchical and complex designs, identification of the appropriate level for tests and full reporting of outcomes
[x]	[ ]	Estimates of effect sizes (e.g. Cohen's *d*, Pearson's *r*), indicating how they were calculated

Our web collection on statistics for biologists contains articles on many of the points above.

Software and code

Policy information about availability of computer code

Data collection	Cryo-EM data were recorded using the EPU (version 1.1.0.30) automated acquisition software package.
Data analysis	Cryo-EM data were processed using the RELION pipeline (versions 2.1 and 3.0), which included motion correction with MOTIONCOR2 (version 1.0.0) and GCTF (version 1.06). Reconstructions were inspected with Chimera (version 1.14) and model coordinates were manipulated using COOT (version 0.9.3). The programs phenix.map_box (for map cropping), phenix.resolve_cryo_em (for density modification), phenix.real_space_refine (for model refinement), Molprobity and EMRinger (for validation), were implemented through the Phenix graphical user interface (versions 1.17.1 and 1.18.2). Structural figures were prepared using Chimera (version 1.14) and ChimeraX (version 1.1).

For manuscripts utilizing custom algorithms or software that are central to the research but not yet described in published literature, software must be made available to editors and reviewers. We strongly encourage code deposition in a community repository (e.g. GitHub). See the Nature Research guidelines for submitting code & software for further information.

Data

Policy information about availability of data

All manuscripts must include a data availability statement. This statement should provide the following information, where applicable:

- Accession codes, unique identifiers, or web links for publicly available datasets
- A list of figures that have associated raw data
- A description of any restrictions on data availability

The sequence of the NωV α coat protein used in the study is from Genbank Accession no.MT875167 as stated in the MS.

All the data supporting the cryo-EM structural work have been deposited in the appropriate databases; specifically the raw data are available from EMPIAR

(accessions 10555 and 10560), the reconstructions are available from EMDB (accessions 11911 and 11830), and the model coordinates are available from the PDB (accessions 7ATA and 7ANM).

All data is freely available.

Field-specific reporting

Please select the one below that is the best fit for your research. If you are not sure, read the appropriate sections before making your selection.

- [x] Life sciences
- [ ] Behavioural & social sciences
- [ ] Ecological, evolutionary & environmental sciences

For a reference copy of the document with all sections, see nature.com/documents/nr-reporting-summary-flat.pdf

Life sciences study design

All studies must disclose on these points even when the disclosure is negative.

Sample size	The research reported concerns molecular analysis. The sample size was therefore determined by measuring such factors as protein concentration, sample volume and uv/vis absorbance as desribed in te MS.
Data exclusions	No data were excluded.
Replication	Multiple preparations of procapsids and capsids were made and the range of yield is cited in the MS.<br><br>The cryo structures reported were derived from 5,426 and 21,395 particle images for the procapsids and capsids, respectively. Full details of the analysis are provide in Table S1.
Randomization	This is not relevant as samples were not allocated into different groups
Blinding	This is not relevant as samples were not allocated into different groups

Reporting for specific materials, systems and methods

We require information from authors about some types of materials, experimental systems and methods used in many studies. Here, indicate whether each material, system or method listed is relevant to your study. If you are not sure if a list item applies to your research, read the appropriate section before selecting a response.

Materials & experimental systems

n/a	Involved in the study	
[ ]	[x]	Antibodies
[x]	[ ]	Eukaryotic cell lines
[x]	[ ]	Palaeontology and archaeology
[x]	[ ]	Animals and other organisms
[x]	[ ]	Human research participants
[x]	[ ]	Clinical data
[x]	[ ]	Dual use research of concern

Methods

n/a	Involved in the study	
[x]	[ ]	ChIP-seq
[x]	[ ]	Flow cytometry
[x]	[ ]	MRI-based neuroimaging

Antibodies

Antibodies used	NωV polyclonal Ab, Target: NωV coat protein, Species: Rabbit<br>Ab6721 (Abcam), Target: Rabbit IgG, Species: Goat
Validation	The primary NωV antibody was validated by testing against plant tissue either expressing or not expressing the NωV capsid protein and against purified preparations of NωV particles of known concentration.
